# Racial and Ethnic Reporting and Representation in US Alzheimer Clinical Trials: A Systematic Review

**DOI:** 10.1001/jamanetworkopen.2026.2427

**Published:** 2026-03-27

**Authors:** Zhuoer Lin, Ruochen Sun, Joseph S. Ross, Kien Lau, Sophia Stumpf, Xi Chen

**Affiliations:** 1 Division of Health Policy and Administration, School of Public Health, University of Illinois Chicago, Chicago; 2 Department of Health Care Management and Economics, The Wharton School, University of Pennsylvania, Philadelphia; 3 Section of General Internal Medicine, Department of Medicine, Yale School of Medicine, New Haven, Connecticut; 4 Department of Health Policy and Management, Yale School of Public Health, New Haven, Connecticut; 5 Yale College, Yale University, New Haven, Connecticut

## Abstract

**Importance:**

Alzheimer disease (AD) disproportionately affects racial and ethnic populations underrepresented in US clinical research, raising concerns about the generalizability of AD trial findings and the evaluation of treatment safety and efficacy for populations most affected by AD.

**Objective:**

To examine patterns and trends in the reporting and representation of patient race and ethnicity in US-based phase 3 AD clinical trials.

**Evidence Review:**

This systematic review examined US-based phase 3 AD drug trials identified through the Trialtrove trial database between 1997 and 2023. Trials were cross-referenced with peer-reviewed publications, ClinicalTrials.gov, pharmaceutical company reports, and conference abstracts. Completed trials were eligible for inclusion if they were designated as phase 3 drug trials targeting AD and recruited patients exclusively in the US. Primary outcomes included reporting of race and ethnicity, the number of racial and ethnic groups reported, and their representation among trial populations. Secondary outcomes included terminology used, reporting of safety or efficacy differences by race and ethnicity, and discussion of racial and ethnic representation in trial reports. Temporal trends in reporting and representation were assessed. Methodologic quality was evaluated using the Quality Rating Scheme for Studies and Other Evidence. Data collection was completed May 2024.

**Findings:**

Among 88 US-based phase 3 AD clinical trials conducted between 1997 and 2023, 71 (80.7%) had publicly available results, including 52 (59.1%) published in peer-reviewed journals. Nearly half of published trials (35 [49.3%]) did not report patient race or ethnicity. Among published trials, reporting was inconsistent and focused predominantly on White (36 [50.7%]) patients, with substantially fewer trials reporting data on Asian or Pacific Islander (11 [15.5%]), Black (20 [28.2%]), Hispanic (13 [18.3%]), or Native American (2 [2.8%]) patients. Median (IQR) enrollment of White patients was 91.3% (87.3%-93.6%), whereas enrollment of underrepresented patient populations was markedly lower, with median (IQR) enrollment of 0.9% (0.6%-1.6%) for Asian or Pacific Islander, 4.5% (3.6%-6.6%) for Black (ethnicity unspecified), 7.2% (3.7%-9.1%) for Black (non-Hispanic), 5.2% (3.1%-6.6%) for Hispanic, and 0.4% (0%-0.8%) for Native American patients. Few trials (3 of 71 [4.2%]) conducted subgroup analyses by race or ethnicity, and none reported detailed subgroup characteristics or safety or efficacy outcomes by patient race and ethnicity. Reporting practices and representation showed little improvement over time.

**Conclusions and Relevance:**

US-based phase 3 AD trials showed substantial gaps in racial and ethnic reporting and representation from 1997 to 2023, limiting the evaluation of treatment safety and efficacy across diverse populations. These findings suggest that stronger reporting standards and more inclusive trial design and recruitment strategies are needed to improve the equity and generalizability of AD trials.

## Key Points

**Question:**

What are the patterns and trends in racial and ethnic reporting and representation in US-based phase 3 Alzheimer disease (AD) clinical trials?

**Findings:**

In this systematic review including 71 published trials with publicly available results from 1997 to 2023, many AD trials did not report patient race or ethnicity, and when reported, practices were inconsistent and largely focused on White patients, with low representation of other racial and ethnic groups. Few trials examined treatment safety or efficacy by patient race or ethnicity; and reporting and representation showed little improvement over time.

**Meaning:**

Findings indicate that persistent gaps in racial and ethnic reporting and underrepresentation in AD trials limit transparency and evaluation of treatment safety and efficacy for populations most affected by AD.

## Teaser

This systematic review analyzes patterns and trends in the reporting and representation of race and ethnicity in US-based phase 3 Alzheimer disease clinical trials from 1997 to 2023.

## Introduction

Alzheimer disease (AD) affects more than 7 million Americans and disproportionately impacts racial and ethnic populations underrepresented in clinical research in the US.^1^ Non-Hispanic Black and Hispanic older adults are nearly twice and 1.5 times as likely, respectively, to have AD than non-Hispanic White older adults^1,2,3,4,5^ yet face substantial barriers to participation in clinical trials.^6,7,8,9^ As the US population ages and becomes more diverse, these disparities are expected to widen, further intensifying the burden of AD and pointing to the urgency of addressing inequities in disease burden and research representation.^1,2,3,4,5^

These growing disparities highlight the need to assess whether emerging treatments are safe and effective across diverse groups.^10,11^ Clinical trials, particularly phase 3 trials, provide the foundation for clinical guidelines and regulatory decisions and are critical for establishing the safety and efficacy of new AD treatments.^6,12,13^ However, phase 3 trials have often fallen short of enrolling racial and ethnic underrepresented populations, including non-Hispanic Black, Hispanic, and Native American individuals, and frequently fail to reflect the racial and ethnic diversity of the US population.^9,11,14,15,16^ Such underrepresentation limits the generalizability of trial findings and may exacerbate disparities in care, as well-documented differences in genetic, behavioral, and clinical factors of AD across racial and ethnic groups can influence therapeutic safety and efficacy.^17,18,19,20,21^ For example, prior research has demonstrated racial and ethnic differences in *APOE ε4* carrier prevalence, burden of vascular and cardiometabolic conditions, health behaviors, health care access and utilization, and clinical presentation of cognitive symptoms, all of which may be associated with AD risk and treatment response.^1,22,23,24^ Despite these concerns, reporting and inclusion by race and ethnicity remain limited and inconsistent.^6^

Previous reviews of AD trials often did not distinguish between trial phases, combined US and non-US trials, or relied primarily on selected bibliographic databases or trial registries, limiting their applicability and policy relevance to the US context.^6,19^ Moreover, the extent to which US-based AD trials have achieved meaningful racial and ethnic representation remains unclear, particularly given rapid demographic shifts in which racial and ethnic underrepresented groups constitute the fastest-growing segments of the older adults population.^11,25^ Focusing on US-based phase 3 trials is therefore critical to ensuring that trial findings are directly relevant to the populations most affected by AD.^9,11,17,25,26^

Using comprehensive trial databases, we characterized the trends to date in how patient race and ethnicity have been reported, analyzed, and represented in US-based phase 3 AD clinical trials from 1997 to 2023. We assessed the extent to which racial and ethnic differences were considered in the trial design and analysis and evaluated changes in reporting and representation over time.

## Methods

This systematic review was determined to be non–human participants research. This study followed the Preferred Reporting Items for Systematic Reviews and Meta-Analyses (PRISMA) reporting guideline.

### Data Sources and Trial Identification

We conducted a systematic review of US-based phase 3 AD clinical trials of drug treatment using a multisource approach. AD trials were identified primarily through the Trialtrove clinical trial database, which provides comprehensive tracking of clinical trials from initiation through completion. Trialtrove aggregates information from a wide variety of sources, including pharmaceutical companies, regulatory agencies, trial registries, conference proceedings, press releases, and peer-reviewed publications.^27,28,29^ The database has been validated in prior research^28,29^ and offers detailed information on trial design, enrollment characteristics, study timelines, and reported outcomes, enabling precise identification, selection, and analysis of clinical trials.^27,28,29^

In this study, Trialtrove was queried to identify all phase 3 AD clinical trials as of April 21, 2023. To ensure completeness and accuracy, identified trials were cross-referenced with PubMed, trial registries, and other public sources. This approach enabled inclusion of both published and unpublished trials and supported comprehensive identification regardless of registration and publication status.

### Eligibility Criteria and Study Selection

Trials were eligible for inclusion if they met the following criteria: (1) designated as phase 3 drug trials; (2) targeted AD; (3) considered as completed; and (4) recruited patients exclusively in the US. Trials were excluded if they were ongoing, planned, terminated, or suspended without publicly available data; were not phase 3 drug trials; or included non-US populations.

The initial search identified 484 phase 3 AD trials. After excluding trials that were open or planned (67 trials), lacked US populations (178 trials), or recruited patients outside the US (136 trials), 103 US-based phase 3 trials remained for further review.

Two reviewers (K.L., S.S.) independently screened 103 trials by examining associated publications, registries, or public sources. We excluded 15 trials that were incomplete with no publicly available records (10 terminated, 1 suspended), not phase 3 (2 trials), or not fully US-based (2 trials) after the review. The final analytic sample included 88 completed US-based phase 3 AD trials.^30,31,32,33,34,35,36,37,38,39,40,41,42,43,44,45,46,47,48,49,50,51,52,53,54,55,56,57,58,59,60,61,62,63,64,65,66,67,68,69,70,71,72,73,74,75,76,77,78,79,80,81,82,83,84,85,86,87,88,89,90,91,92,93,94,95,96,97,98,99,100,101,102,103,104,105,106,107,108,109,110,111,112,113,114,115,116,117^ The sample selection process is presented in eFigure 1 in Supplement 1, and the distribution of trials over publication years is given in eFigure 2 in Supplement 1. A list of the 88 trials, together with their quality ratings, is provided in the eAppendix and cited in the eReferences in Supplement 1. The quality of evidence was assessed using the Quality Rating Scheme for Studies and Other Evidence.

### Data Extraction and Quality Assurance

We followed a 2-stage process to extract data on patient race and ethnicity reporting and representation. In the first stage, 2 reviewers (K.L., S.S.) independently extracted all reported racial and ethnic data from available sources. For each trial with published reports, the data source and publication date were documented. Publication dates in our sample ranged from 1997 to 2023. In the second stage, the reviewers cross-checked their extracted data and resolved discrepancies through consensus. A third reviewer (Z.L.) then performed a final quality check. Data collection was completed in May 2024.

### Outcome Measures

The primary outcomes were measures of patient racial and ethnic reporting and representation in US-based phase 3 AD trials, including whether race and ethnicity were reported, the number of racial and ethnic groups reported, the terminology used, and the proportion of patients from each racial and ethnic group. Secondary outcomes included whether trials reported sample characteristics, subgroup analyses or differences in safety or efficacy by race and ethnicity, and whether trial reports discussed the racial and ethnic representation of enrolled patients.

Terminology used to describe racial and ethnic groups varied widely and was often inconsistent with existing reporting guidelines.^118,119,120,121,122^ Because Hispanic ethnicity was frequently unspecified, racial and ethnic categories were analyzed separately for Black (ethnicity unspecified) and Black (non-Hispanic) patients as well as for White (ethnicity unspecified) and White (non-Hispanic) patients. Additional categories included Asian or Pacific Islander, Hispanic, and Native American patients, as documented in trial reports.

### Statistical Analysis

Trials were considered published if trial data appeared in peer-reviewed journals, ClinicalTrials.gov, pharmaceutical reports, or conference abstracts. Our analyses focused on all published trials, with additional emphasis on trials reported in peer-reviewed publications. When multiple reports were available for a single trial, the report containing the most complete information was selected.

Descriptive statistics were used to summarize trial characteristics, reporting practices, and patient composition. Time trends in racial and ethnic reporting were assessed by estimating changes over time in the proportion of trials reporting race and ethnicity and the number of racial and ethnic groups reported. Time trends in representation were evaluated by examining changes in the percentage of enrolled patients across racial and ethnic categories over time. Linear regression models with robust standard errors were used to estimate temporal trends, with statistical significance assessed at a 2-sided 5% level. Analyses were conducted using R, version 4.5.0 (R Project for Statistical Computing), and Stata, version 17.0 (StataCorp LLC).

## Results

The Table presents the characteristics of US-based phase 3 AD clinical trials. Of 88 phase 3 AD clinical trials reported between 1997 and 2023,^30,31,32,33,34,35,36,37,38,39,40,41,42,43,44,45,46,47,48,49,50,51,52,53,54,55,56,57,58,59,60,61,62,63,64,65,66,67,68,69,70,71,72,73,74,75,76,77,78,79,80,81,82,83,84,85,86,87,88,89,90,91,92,93,94,95,96,97,98,99,100,101,102,103,104,105,106,107,108,109,110,111,112,113,114,115,116,117^ 71 (80.7%) had published data available,^30,33,34,35,36,37,40,43,44,46,47,50,51,52,55,56,57,58,59,60,61,62,63,66,67,69,70,71,72,73,74,75,76,77,78,79,81,82,83,84,85,86,87,88,89,90,91,92,93,94,95,96,97,98,100,101,102,103,104,105,107,108,109,110,111,112,113,114,115,116,117^ while 17 (19.3%) had no publications.^31,32,38,39,41,42,45,48,49,53,54,64,65,68,80,99,106^ In total, 52 trials (59.1%) were published in peer-reviewed journals^30,33,34,35,37,43,44,50,51,55,56,57,58,59,60,61,62,66,67,70,72,74,75,76,77,78,79,81,82,83,84,85,87,88,89,90,91,94,95,96,97,98,100,101,108,109,110,111,112,113,115,117^ and the remaining trials reported results through ClinicalTrials.gov, pharmaceutical company reports, or conference abstracts.^36,40,46,47,52,63,69,71,73,86,92,93,102,103,104,105,107,114,116^

**Table.** Characteristics, Racial and Ethnic Reporting, and Representation of Phase 3 Alzheimer Disease Clinical Trials in the US, 1997-2023

Characteristic	Trials	
	All trials (n = 88)	Trials with peer-reviewed publications (n = 52)
Data source, No. (%)		
Peer-reviewed publications	52 (59.1)	52 (100)
ClinicalTrials.gov registries	8 (9.1)	0
Trial reports of the pharmaceutical companies	2 (2.3)	0
Conference abstracts	9 (10.2)	0
No publication	17 (19.3)	0
Year of publication among published, No. (%)^a^		
2000 or earlier	5 (7.0)	5 (9.6)
2001-2005	24 (33.8)	19 (36.5)
2006-2010	29 (40.8)	20 (38.5)
2011-2015	9 (12.7)	7 (13.5)
2016-2023	4 (5.6)	1 (1.9)
Sample size, median (IQR)^a^	191 (85-433)	324 (134-525)
Race or ethnicity reported, No. (%)^a^		
None	35 (49.3)	18 (34.6)
Asian or Pacific Islander	11 (15.5)	10 (19.2)
Black (ethnicity unspecified)	10 (14.1)	9 (17.3)
Black (non-Hispanic)	10 (14.1)	10 (19.2)
Hispanic	13 (18.3)	12 (23.1)
Native American	2 (2.8)	1 (1.9)
White (ethnicity unspecified)	25 (35.2)	23 (44.2)
White (non-Hispanic)	11 (15.5)	11 (21.2)
No. of racial and ethnic groups reported, median (IQR)^a^	1 (0-3.0)	1 (0-3.5)
Percentage of Asian or Pacific Islander patients, median (IQR)^b^	0.9 (0.6-1.6)	0.9 (0.6-1.6)
Percentage of Black (ethnicity unspecified) patients, median (IQR)^b^	4.5 (3.6-6.6)	4.6 (4.1-6.6)
Percentage of Black (non-Hispanic) patients, median (IQR)^b^	7.2 (3.7-9.1)	7.2 (3.7-9.1)
Percentage of Hispanic patients, median (IQR)^b^	5.2 (3.1-6.6)	5.6 (2.5-6.6)
Percentage of Native American patients, median (IQR)^b^	0.4 (0-0.8)	0.8 (0.8-0.8)
Percentage of White (ethnicity unspecified) patients, median (IQR)^b^	91.3 (87.3-93.6)	91.3 (87.3-93.6)
Percentage of White (non-Hispanic) patients, median (IQR)^b^	84.1 (80.1-93.4)	84.1 (80.1-93.4)
Reporting sample characteristics by racial and ethnic groups, No. (%)^a^		
No	71 (100)	52 (100)
Yes	0	0
Reporting detailed trial outcomes (eg, safety, efficacy) by racial and ethnic groups, No. (%)^a^		
No	71 (100)	52 (100)
Yes	0	0
Analyzing or discussing heterogeneity or differences across racial and ethnic groups, No. (%)^a^		
No	68 (95.8)	49 (94.2)
Yes	3 (4.2)	3 (5.8)
Discussing the racial and ethnic representation of enrolled trial patients, No. (%)^a^		
No	71 (100)	52 (100)
Yes	0	0

^a^ Among trials with any published data, n = 71 for any publications, and n = 52 for peer-reviewed publications.

^b^ Among trials with reported data on race and ethnicity. The sample size for each reported race and ethnicity is provided in the table.

### Racial and Ethnic Reporting in AD Trials

Among 71 published trials, nearly half (35 [49.3%]) did not report any data on patient race or ethnicity.^35,36,46,50,51,52,56,58,59,62,63,69,70,71,73,75,79,83,86,88,89,92,93,96,101,102,103,104,105,107,109,113,114,115,116^ Only 25 trials (35.2%) reported data on White patients (ethnicity unspecified),^25,30,33,34,37,40,44,47,57,61,66,74,76,78,81,84,85,87,91,94,95,97,100,110,117^ and 11 trials (15.5%) on White (non-Hispanic) patients.^43,55,60,67,72,77,82,98,108,111,112^ Reporting of other racial and ethnic groups was limited. Specifically, only 11 trials (15.5%) reported data on Asian or Pacific Islander patients,^47,55,72,74,82,87,91,98,100,108,112^ 10 trials (14.1%) on Black (ethnicity unspecified) patients,^33,47,57,61,66,74,87,91,94,100^ 10 trials (14.1%) on Black (non-Hispanic) patients,^43,55,67,72,77,82,98,108,111,112^ 13 trials (18.3%) on Hispanic patients,^43,47,55,57,60,67,72,77,82,98,108,111,112^ and 2 trials (2.8%) on Native American patients^47,72^ (Table).

Reporting proportions were modestly higher among trials published in peer-reviewed journals; however, substantial underreporting persisted. The median (IQR) number of patient racial and ethnic groups reported was 1 (0-3.0) across all published trials, and 1 (0-3.5) among peer-reviewed trials, with most trials reporting only the number or percentage of White patients (data presented in the next subsection and in the Table).

Trials differed in whether they reported race, ethnicity, or both; whether Hispanic ethnicity was clearly distinguished from race; and how racial and ethnic categories were defined (eTable in Supplement 1). No trials reported sample characteristics (eg, demographic or clinical characteristics) stratified by race or ethnicity. Only 3 of 71 trials (4.2%) conducted any subgroup analyses by race or ethnicity, and none reported detailed treatment efficacy or safety outcomes stratified by racial or ethnic group or provided sufficient information to assess differential treatment effects (Table).

### Racial and Ethnic Representation in AD Trials

When racial and ethnic composition was reported, White patients constituted the majority of enrolled populations. The median (IQR) percentage of White patients was 91.3% (87.3%-93.6%) among trials reporting White race and 84.1% (80.1%-93.4%) among trials reporting non-Hispanic White ethnicity.

In contrast, patients from racial and ethnic underrepresented groups accounted for a small proportion of trial enrollment. Median (IQR) enrollment populations were 0.9% (0.6%-1.6%) for Asian or Pacific Islander, 4.5% (3.6%-6.6%) for Black (ethnicity unspecified), 7.2% (3.7%-9.1%) for Black (non-Hispanic), 5.2% (3.1%-6.6%) for Hispanic, and 0.4% (0%-0.8%) for Native American patients. No trials acknowledged underrepresentation in enrollment, discussed its implications for generalizability, or proposed strategies to improve representation (Table).

### Trends in Racial and Ethnic Reporting and Representation

**Figure 1.**
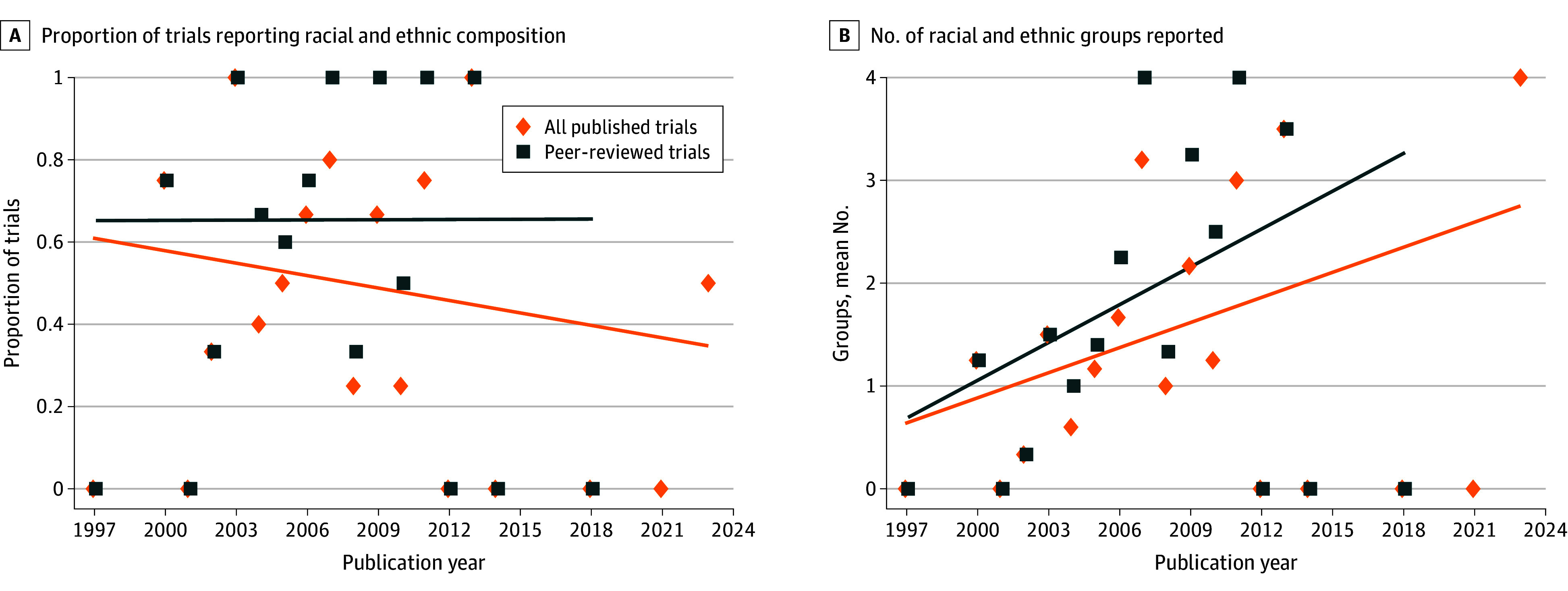
Scatter Plots Showing Trends in Racial and Ethnic Reporting Among US-Based Phase 3 Alzheimer Disease Trials, 1997-2023 Linear time trends were fitted respectively for all published trials. No statistically significant linear trends were observed.

Figure 1 illustrates trends in racial and ethnic reporting. Over time, the proportion of trials reporting any patient race or ethnicity data did not improve, although the number of racial and ethnic groups reported slightly increased. Among all published trials, there was a nonsignificant decline in the proportion reporting racial and ethnic data (slope, −1.0% [95% CI, −3.4% to 1.4%]), whereas reporting remained stable among trials published in peer-reviewed journals (slope, 0.02% [95% CI, −3.7% to 3.7%]). The number of racial and ethnic groups reported showed an increase among peer-reviewed trials, although this trend was not statistically significant (slope, 0.12 [95% CI, −0.01 to 0.26]).

**Figure 2.**
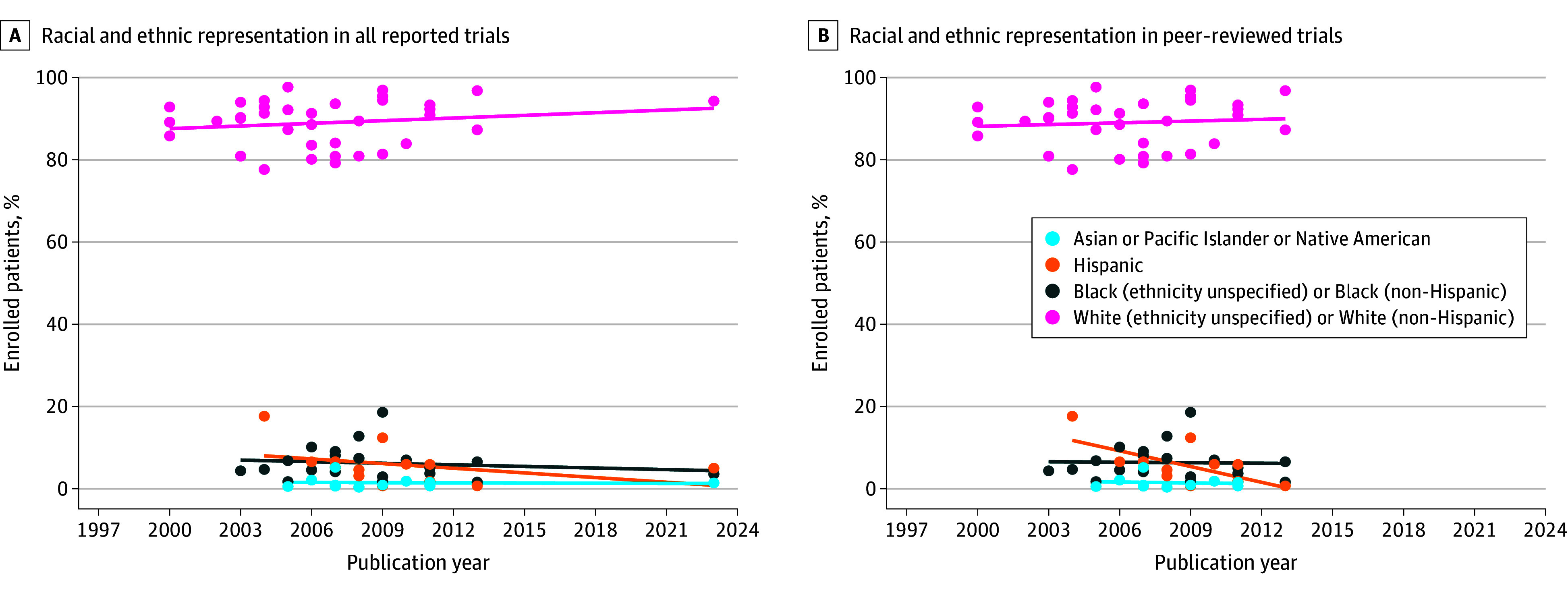
Scatter Plots Illustrating Trends in Racial and Ethnic Representation Among US-Based Phase 3 Alzheimer Disease Trials, 1997-2023 Linear time trends were fitted for each racial and ethnic group. No statistically significant linear trends were observed, except for a significant decline in Hispanic representation in peer-reviewed trials.

Figure 2 and eFigure 3 in Supplement 1 present trends in racial and ethnic group representation among trial patients. White patients consistently constituted nearly 90% of trial enrollment over time, with a slight but nonsignificant increasing trend. In contrast, enrollment of racial and ethnic underrepresented groups remained persistently low, with no evidence of improvement over time. Among peer-reviewed trials, Hispanic representation showed a modest and statistically significant decline (slope, −1.3% [95% CI, −2.4% to −0.19%]).

## Discussion

This systematic review of US-based phase 3 AD clinical trials reported between 1997 and 2023 reveals persistent and substantial gaps in both reporting of patient race and ethnicity and representation. Despite decades of recognition that AD disproportionately affects certain racial and ethnic groups, including non-Hispanic Black and Hispanic populations,^1,2^ nearly half of trials (49.3%) failed to report any information on patient race or ethnicity, with little evidence of improvement over time. Among trials that did report these data, enrollment of racial and ethnic underrepresented populations remained consistently low, and few trials conducted subgroup analyses by patient race or ethnicity. As a result, the ability to assess treatment safety or efficacy across diverse populations remains constrained.^6,11,123^ These patterns raise important concerns about the generalizability of trial findings and the extent to which current evidence can support equitable treatment decisions for populations most affected by AD.^6,17^

Inadequate reporting of race and ethnicity represents a fundamental limitation of current clinical evidence to inform AD treatment. Even when demographic information was reported, we found that practices were often inconsistent, with wide variation in terminology, categorization, and distinctions between race and ethnicity. Reporting frequently focused on White patients, with limited attention to other racial and ethnic groups and little clarity regarding how categories were defined or used in analyses. These inconsistencies limit transparency and hinder meaningful comparisons across trials. More importantly, incomplete reporting constrains the ability to assess generalizability and evaluate whether treatment safety and efficacy may differ across populations, despite well-documented differences in genetic risk profiles, comorbidity burden, and social determinants of health across racial and ethnic groups.^1,17,20,21,124^ The persistence of these reporting gaps highlights the importance of further strengthening and consistently implementing race and ethnicity reporting standards, including recent initiatives by federal agencies and academic journals.^118,119,120,121,122^

Our findings reveal a persistent mismatch between AD disease burden and trial enrollment. In the US, non-Hispanic Black older adults experience nearly twice the prevalence of AD compared with non-Hispanic White older adults, and Hispanic older adults face approximately 50% higher prevalence than non-Hispanic White older adults.^1^ Notably, in 2020, non-Hispanic Black individuals accounted for 17.5% of all people living with AD, while Hispanic individuals accounted for 11.7%, with percentages projected to increase to 24.5% for Black individuals and 26.8% for Hispanic individuals by 2060.^125^ Yet phase 3 AD trials have continued to enroll disproportionately few patients from these groups. From 1997 to 2023, we showed that median enrollment remained 4.5% to 7.2% for Black patients, 5.2% Hispanic patients, and less than 1% for Native American patients, whereas White patients consistently comprised 91.3% of trial populations. This marked imbalance has important implications for both equity and scientific validity. When trial populations do not reflect individuals most affected by AD, the resulting evidence may inadequately capture variation in treatment response, adverse events, and clinical practice effectiveness.^6^ This concern is particularly salient for AD, given well-established differences in disease progression, comorbid conditions, and access to diagnosis and care across populations.^1,11^

The limited progress observed in reporting and representation likely reflects broader structural and trial-level barriers.^6,7,8,9,10,11,14,19,123,126,127,128,129,130,131,132,133,134^ Restrictive eligibility criteria, including exclusions related to comorbidities or cognitive screening thresholds, may disproportionately exclude individuals from populations with higher disease burden.^9,19,135^ Trial sites are often concentrated in locations that are less accessible to individuals living in disadvantaged or underserved communities, further limiting participation by racial and ethnic underrepresented groups. In addition, investigators may face practical constraints, including limited time, funding, expertise, and access to culturally appropriate resources, which may challenge effective engagement with underrepresented communities.^136^ Recruitment strategies also often lack partnerships with community organizations and fail to address limited awareness of AD and research opportunities, language barriers, cultural relevance, stigma, logistical challenges, and longstanding mistrust of research institutions.^6,7,8,9,10,11,14,19,123,126,127,128,129,130,131,132,133^ These factors, together, help explain why improvements in enrollment and reporting have remained limited despite increased attention to diversity in clinical research.

Addressing these challenges will require coordinated multilevel efforts.^6,10,11,14,19,123^ First, standardized race and ethnicity reporting, with consistent definitions and mandatory registry fields, provides a necessary foundation for improving transparency, comparability, and accountability.^17,19,21^ This reporting should follow established guidance from federal agencies and journals, including separate collection of race and ethnicity, consistent use of predefined categories, explicit distinction of Hispanic ethnicity from race, allowance for multiple racial identifies, and clear indication of missing or unknown responses.^118,119,120,121,122^ Consistent application of these standards across trial registries and publications would improve comparability and interpretability across AD trials. Second, beyond reporting, meaningful progress in representation will depend on intentional trial design choices, including more inclusive eligibility criteria, broader and more diverse site selection, and recruitment strategies that actively engage racial and ethnic populations disproportionately affected by AD.^7,19^ Financial supports, such as transportation assistance or patient compensation, may help mitigate socioeconomic barriers that disproportionately limit participation among underrepresented populations.^19,26,129^ In addition, partnerships with local advocacy groups and health care professionals, use of bilingual and culturally concordant research staff, and tailored outreach materials for underserved communities may further reduce barriers, strengthen trust, and improve accessibility.^7,18,20,21,129,131^ These approaches should be adopted more widely to ensure inclusive and generalizable AD research.^6,10,11,14,19,123^ Finally, policy initiatives, such as those proposed in the bipartisan Equity in Neuroscience and Alzheimer Clinical Trials (ENACT) Act, may play an important role in addressing these disparities by expanding outreach, strengthening recruitment infrastructure, and promoting a more representative clinical research workforce.^7,137^

### Strengths and Limitations

This study has several strengths. First, by leveraging multiple complementary data sources, including Trialtrove, PubMed, ClinicalTrials.gov, conference abstracts, and pharmaceutical reports, we identified a comprehensive list of US-based AD trials conducted between 1997 and 2023. Second, our exclusive focus on US-based phase 3 trials elevated both clinical and policy relevance, as these trials provide the highest level of evidence for treatment efficacy and play a central role in regulatory decision-making for the US population. Third, our assessment of racial and ethnic reporting and representation was both thorough and comprehensive. We examined not only whether demographic data were reported but also how they were reported and the quality of reporting. Additionally, by analyzing trends over time, we identified persistent gaps and highlighted the need for more systematic oversight and regulatory efforts.

This study also has limitations. Although our focus on US-based trials strengthens the relevance for domestic US policy and practice, the study excluded large multinational trials in which underrepresentation is also a substantial concern. The issue of racial and ethnic underrepresentation persists even in broader global trials.^11^ Second, trials that fail to report any racial or ethnic data may be more likely to have enrolled few patients from racial and ethnic underrepresented groups, potentially leading to underestimation of the full extent of these disparities. Finally, although we discussed several potential contributing factors, the underlying causes of underreporting and underrepresentation, as well as the reasons for their persistence over time, warrant further investigation. Future research, particularly using mixed methods, is needed to better understand the mechanisms and identify effective interventions.

## Conclusions

The findings of this systematic review highlight the persistent challenges in providing equitable access to clinical research and the critical need for more inclusive and representative AD trials. To ensure that the benefits of emerging AD treatments are equitably distributed, clinical trials must better reflect the diverse demographics of the US population. Effective and persistent efforts are needed to reduce health disparities and improve outcomes for all individuals affected by AD.

## Notes

Supplement 1. **eFigure 1.** Study Sample Selection Process **eFigure 2.** Number of US-Based Phase III Alzheimer’s Disease Trials Over Publication Years, 1997-2023 **eFigure 3.** Trends in Representation of All Reported Racial and Ethnic Groups Among US-Based Phase III Alzheimer’s Disease Trials, 1997-2023 **eTable.** Terminology Used for Racial and Ethnic Group Reporting Among All Published US-Based Phase III Alzheimer’s Disease Trials, 1997-2023 **eAppendix.** List of 88 US-Based Phase III Alzheimer’s Disease Trials Identified From the Trialtrove Database and Quality Ratings, 1997-2023
eReferences.

Supplement 2.
Data Sharing Statement
