# Supplementary material for: Racial and Ethnic Reporting and Representation in US Alzheimer Clinical Trials: A Systematic Review
Source: JAMA Netw Open. 2026 Mar 27;9(3):e262427. doi: 10.1001/jamanetworkopen.2026.2427 (PMC13032159; doi:10.1001/jamanetworkopen.2026.2427)
Supplement: Supplement 1. — eFigure 1. Study Sample Selection Process eFigure 2. Number of US-Based Phase III Alzheimer’s Disease Trials Over Publication Years, 1997-2023 eFigure 3. Trends in Representation of All Reported Racial and Ethnic Groups Among US-Based Phase III Alzheimer’s Disease Trials, 1997-2023 eTable. Terminology Used for Racial and Ethnic Group Reporting Among All Published US-Based Phase III Alzheimer’s Disease Trials, 1997-2023 eAppendix. List of 88 US-Based Phase III Alzheimer’s Disease Trials Identified From the Trialtrove Database and Quality Ratings, 1997-2023 eReferences. [file jamanetwopen-e262427-s001.pdf]

Supplementary Online Content

Lin Z, Sun R, Ross JS, Lau K, Stumpf S, Chen X. Racial and ethnic reporting and representation in US Alzheimer clinical trials: a systematic review. *JAMA Netw Open*. 2026;9(3):e262427. doi:10.1001/jamanetworkopen.2026.2427

eFigure 1. Study Sample Selection Process
eFigure 2. Number of US-Based Phase III Alzheimer's Disease Trials Over Publication Years, 1997-2023
eFigure 3. Trends in Representation of All Reported Racial and Ethnic Groups Among US-Based Phase III Alzheimer's Disease Trials, 1997-2023
eTable. Terminology Used for Racial and Ethnic Group Reporting Among All Published US-Based Phase III Alzheimer's Disease Trials, 1997-2023
eAppendix. List of 88 US-Based Phase III Alzheimer's Disease Trials Identified From the Trialtrove Database and Quality Ratings, 1997-2023
eReferences.

This supplementary material has been provided by the authors to give readers additional information about their work.

© 2026 Lin Z et al. *JAMA Network Open.*

eFigure 1. Study Sample Selection Process

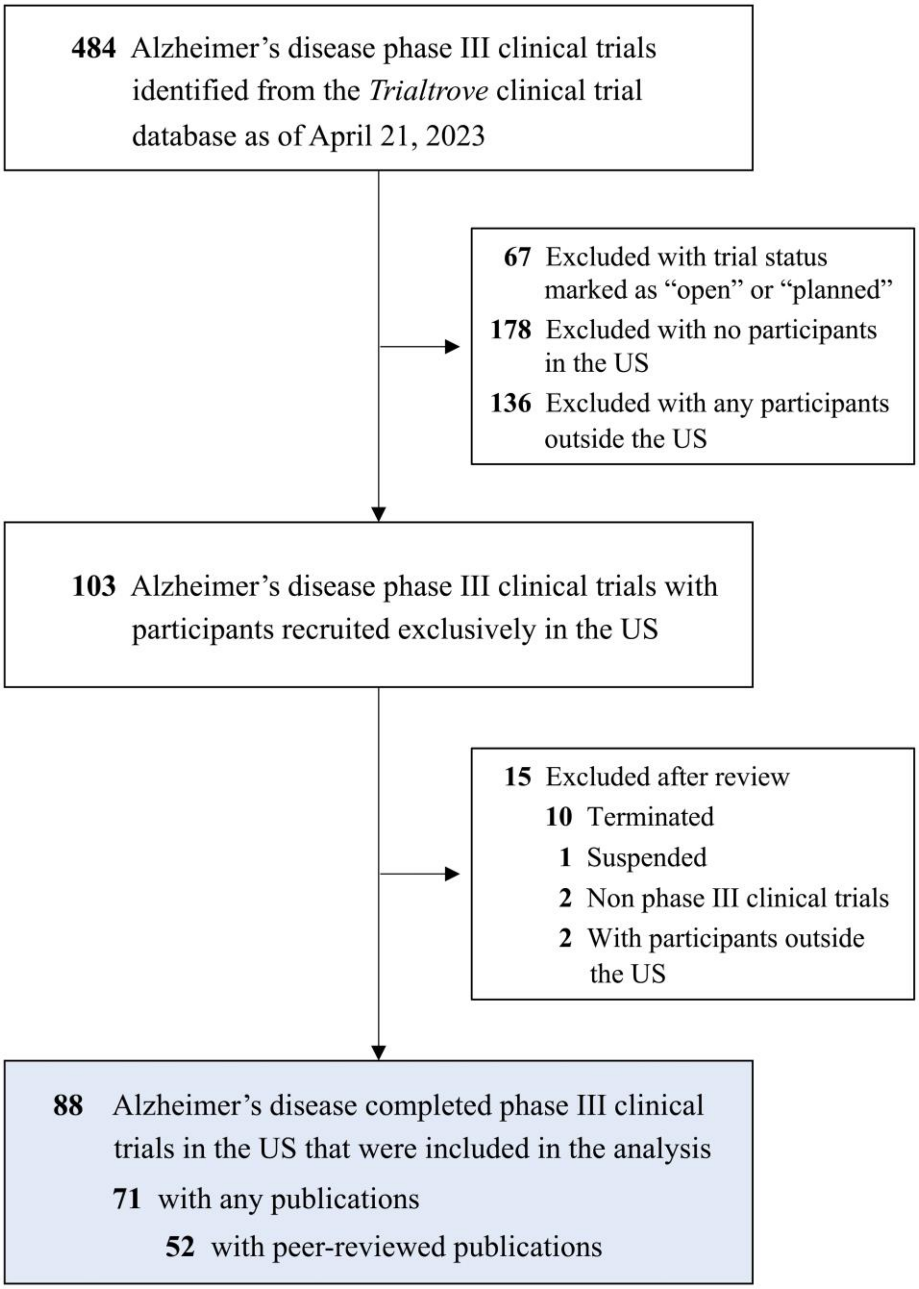

© 2026 Lin Z et al. *JAMA Network Open*.

eFigure 2. Number of US-Based Phase III Alzheimer's Disease Trials Over Publication Years, 1997-2023

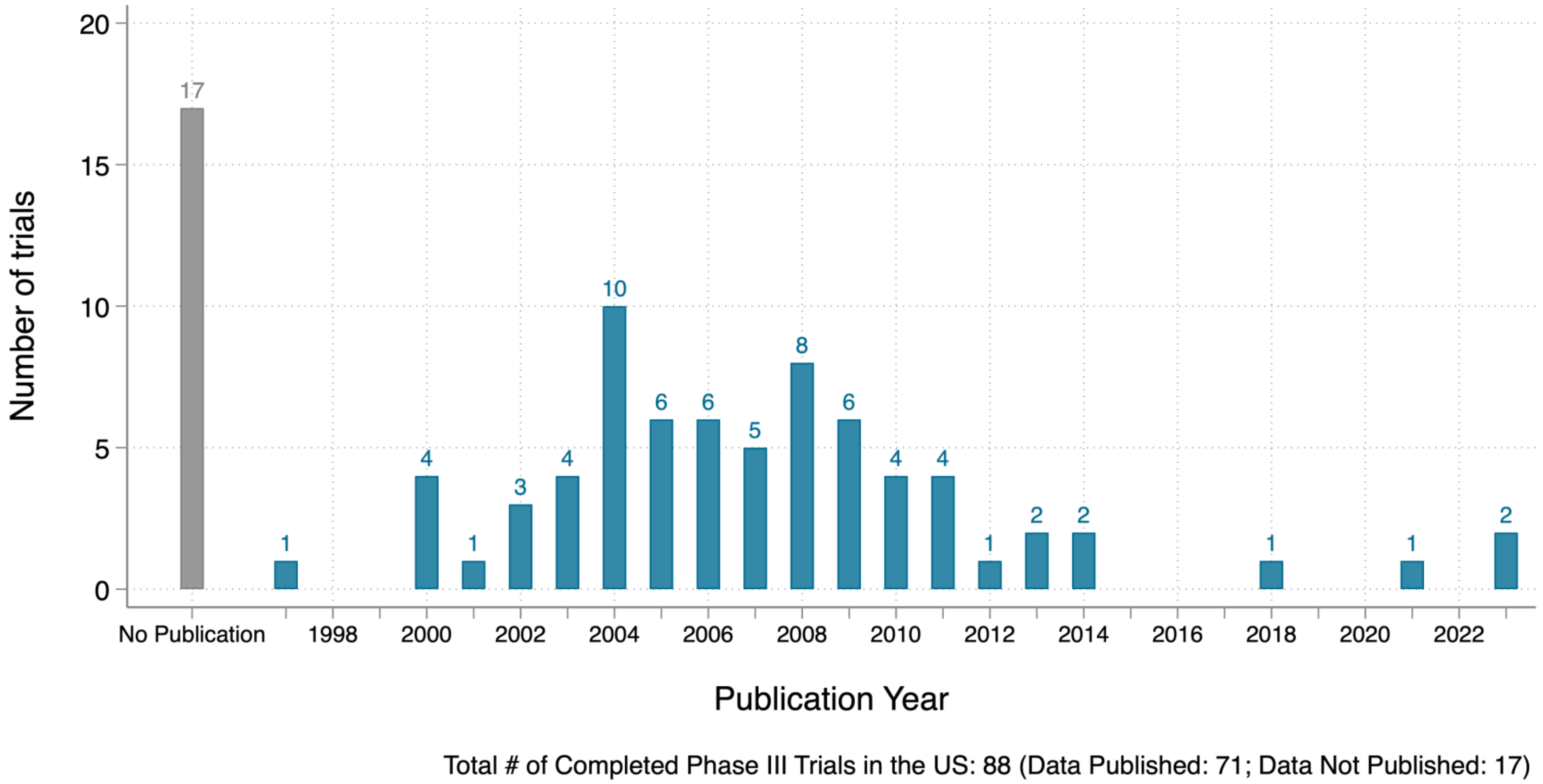

Notes: In the figure, the gray bar represents the number of trials with no publications, and the blue bars represent the number of trials published in each year.

© 2026 Lin Z et al. *JAMA Network Open*.

eFigure 3. Trends in Representation of All Reported Racial and Ethnic Groups Among US-Based Phase III Alzheimer's Disease Trials, 1997-2023

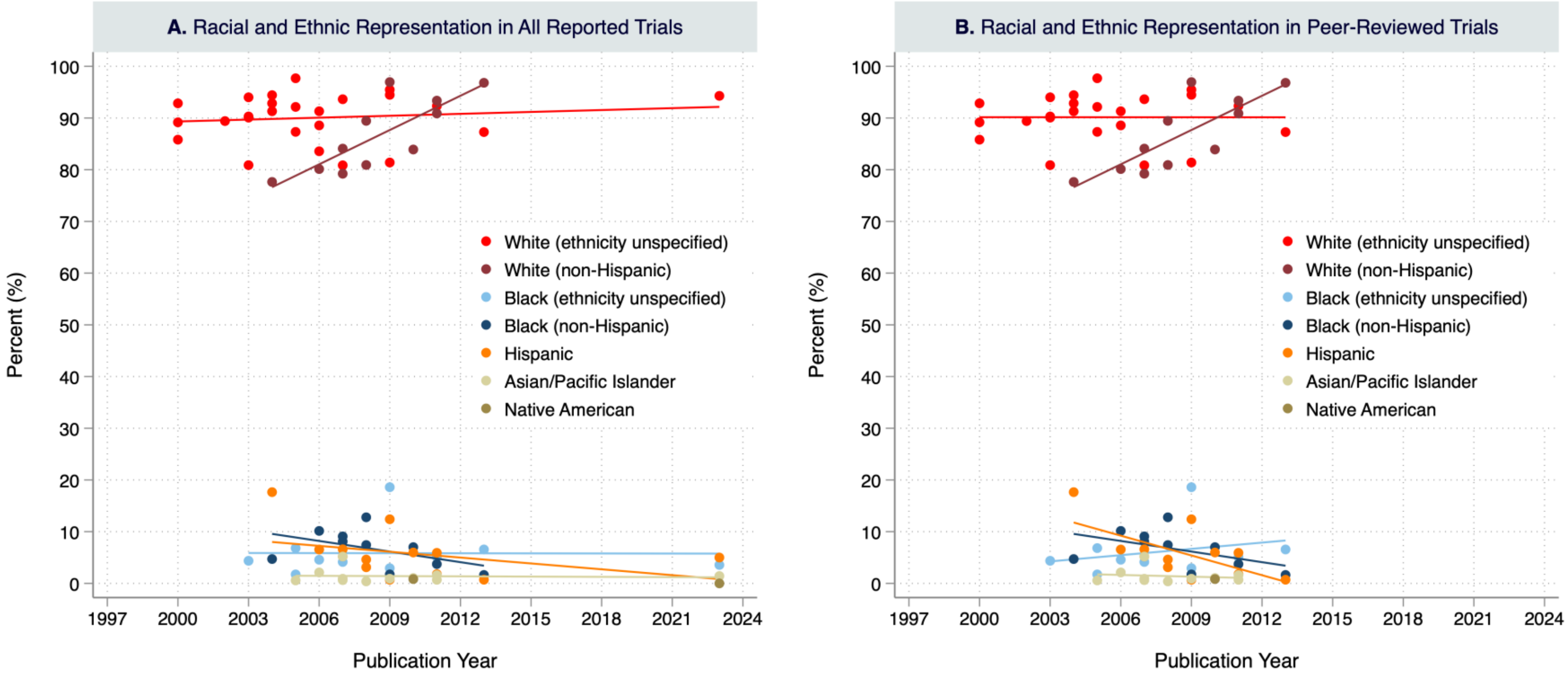

Notes: Panels A and B present the racial and ethnic representation among all published trials (Panel A) and peer-reviewed trials (Panel B). Each dotted points represent the percentage of patients of certain race and ethnicity in each clinical trial published with reported data on that race and ethnicity. Red dots represent the reported percentages of White (ethnicity unspecified) patients; dark red dots represent the reported percentages of White (non-Hispanic) patients; light blue dots represent the reported percentages of Black (ethnicity unspecified) patients; dark blue dots represent the reported percentages of Black (non-Hispanic) patients; orange dots represent the reported percentages of Hispanic patients; light green dots represent the reported percentages of Asian or Pacific Islander patients; and dark green dots represent the reported percentages of Native American patients. Linear time trends were fitted for each racial and ethnic groups with consistent colors as the dotted points. Linear trends for Native American patients were not fitted because only one clinical trial had reported data on the percentage of Native Americans.

© 2026 Lin Z et al. *JAMA Network Open*.

eTable. Terminology Used for Racial and Ethnic Group Reporting Among All Published US-Based Phase III Alzheimer's Disease Trials, 1997-2023

	**No. of studies (%)**	
**Terms used in racial and ethnic reporting**	**Trials with publications (n=71)**	**Trials with peer-reviewed publications (n=52)**
No Race/Ethnicity Reported	35 (49.3)	18 (34.6)
White	10 (14.1)	10 (19.2)
Caucasian	2 (2.8)	1 (1.9)
Minorities	1 (1.4)	1 (1.9)
White, Other	1 (1.4)	1 (1.9)
Caucasian, Hispanic/Latino	1 (1.4)	1 (1.9)
White, African American or Other	1 (1.4)	1 (1.9)
White, Black, Other	1 (1.4)	1 (1.9)
White, Hispanic, Black	1 (1.4)	1 (1.9)
Caucasian, Black, Asian	1 (1.4)	1 (1.9)
Caucasian, Black, Other	1 (1.4)	1 (1.9)
White/Caucasian, Black, Other	1 (1.4)	1 (1.9)
African American, Caucasian, Other	1 (1.4)	1 (1.9)
White, Black, Hispanic, Other	2 (2.8)	2 (2.8)
White, African-American, Hispanic, Other	1 (1.4)	1 (1.9)
White, Black, Asian/Pacific Islander, Hispanic/Latino	1 (1.4)	1 (1.9)

© 2026 Lin Z et al. *JAMA Network Open*.

White, African-American, Asian/Pacific Islander, Other	1 (1.4)	1 (1.9)
White, Black/African American, Hispanic/Latino, Not Hispanic/Latino+	1 (1.4)	1 (1.9)
White, Black, Hispanic, Asian, Other	2 (2.8)	2 (3.8)
Unknown, Asian, Black, White, more than 1 race	1 (1.4)	1 (1.9)
Caucasian, African-American, Hispanic, Asian, Other	1 (1.4)	1 (1.9)
Caucasian, Hispanic, African American, Asian, Other	1 (1.4)	1 (1.9)
Caucasian (White), Non-Caucasian, Black, Asian, Other	1 (1.4)	1 (1.9)
African-American, Asian, White (non-Hispanic), White (Hispanic), Native American	1 (1.4)	1 (1.9)
American Indian or Alaska Native, Asian, Native Hawaiian or Other Pacific Islander, Black or African American, White, More than one race, Unknown or not reported, Hispanic	1 (1.4)	0 (0.0)

Notes: Terms are ordered by the number of racial and ethnic groups reported. All terminology shown reflects the exact wording used in the original publications.

© 2026 Lin Z et al. *JAMA Network Open*.

eAppendix. List of 88 US-Based Phase III Alzheimer's Disease Trials Identified From the Trialtrove Database and Quality Ratings, 1997-2023

Trialtrove TrialID	Trialtrove Trial Title	Trialtrove Trial Phase	Trialtrove Trial Countries	Publication Type	Rating [a]
4007	Study of Melatonin: Sleep Problems in Alzheimer's Disease[1]	III	United States	Peer-Reviewed Publication	1
4013	Ginkgo Biloba Prevention Trial in Older Individuals (Ginkgo Evaluation of Memory Study, GEM Study)[2]	III	United States	Peer-Reviewed Publication	1
4018	High Dose Supplements to Reduce Homocysteine and Slow the Rate of Cognitive Decline in Alzheimer's Disease (Vitamins to slow Alzheimer's - VITAL)[3]	III	United States	Peer-Reviewed Publication	1
4022	Comparison of Citalopram, Cerphenazine and Placebo for the Acute Treatment of Psychosis and Behavioral Disturbances in Hospitalized, Demented Patients[4]	III	United States	Peer-Reviewed Publication	1
4029	Alzheimer's Disease Prevention Trial. A Multi-Center, Randomized, Double-Blind Placebo Controlled Trial of Estrogens to Prevent Alzheimer's Disease and Loss of Memory in Women[5]	III	United States	Peer-Reviewed Publication	1
4030	Placebo Controlled Evaluation of Galantamine in the Treatment of Alzheimer's Disease: Safety and Efficacy Under a Slow-Titration Regimen[6]	III	United States	Peer-Reviewed Publication	1
4032	Alzheimer's Disease Anti-Inflammatory Prevention Trial (ADAPT): A Randomized, Double-Blind, Placebo Controlled, Parallel Assignment, Efficacy Study[7]	III	United States	Peer-Reviewed Publication	1
4035	A Multicenter Trial of Prednisone in Alzheimer's Disease[8]	III	United States	Peer-Reviewed Publication	1
4036	Agitation in Alzheimer's Disease: A Randomized, Placebo-Controlled Clinical Trial[9]	III	United States	Peer-Reviewed Publication	1

© 2026 Lin Z et al. *JAMA Network Open*.

4040	A Multicenter, Double-Blind, Placebo-Controlled Study of Estrogen Replacement Therapy in Patients with Mild to Moderate Alzheimer's Disease[10]	III	United States	Peer-Reviewed Publication	1
4044	A Multi-Center, Randomized, Double-Blind, Placebo-Controlled Trial of Simvastatin to Slow the Progression of Alzheimer's Disease[11]	III	United States	Peer-Reviewed Publication	1
4045	Efficacy and Safety of a Flexible Dose of Risperidone Versus Placebo in the Treatment of Psychosis of Alzheimer's Disease[12]	III	United States	Peer-Reviewed Publication	1
4058	Anti-Inflammatory Treatment for Age-Associated Memory Impairment: A Double-Blind Placebo-Controlled Trial[13]	III	United States	Peer-Reviewed Publication	1
4068	A Randomized, Double-Blind, Placebo-Controlled Trial of Valproate to Attenuate the Progression of Alzheimer's Disease (AD)[14]	III	United States	Peer-Reviewed Publication	1
4078	Memantine in Moderate-to-Severe Alzheimer's Disease[15]	III	United States	Peer-Reviewed Publication	1
4079	A controlled trial of selegiline, alpha-tocopherol, or both as treatment for Alzheimer's disease. The Alzheimer's Disease Cooperative Study[16]	III	United States	Peer-Reviewed Publication	1
4223	A Phase III Study of ABP-124 for Alzheimer's Disease[17]	III	United States	No Publication	1
4256	Anti-Inflammation in AD: PET Imaging Supplement[18]	III	United States	Trial Report from Clinicaltrials.gov	1
7842	Reduction of Behavioral Disturbances and Caregiver Distress by Galantamine in Patients with Alzheimer's Disease[19]	III	United States	Peer-Reviewed Publication	1
8722	Galantamine ER Open Label Rapid Dose Escalation Trial in Alzheimer's Disease[20]	III	United States	Peer-Reviewed Publication	1
10858	A Randomized, Double-Blind, Placebo-Controlled Evaluation of the Safety and Efficacy of Memantine in	III	United States	Peer-Reviewed Publication	1

© 2026 Lin Z et al. *JAMA Network Open*.

	Patients with Mild to Moderate Dementia of the Alzheimer's Type[21]				
11944	Galantamine in the Treatment of Moderate to Advanced Alzheimer's Disease: Clinical and Pharmacoeconomic Outcomes in the Assisted Living Environment[22]	III	United States	Conference Abstract	1
12375	Galantamine Continuation Versus Interruption Trial: Study 1[23]	III	United States	Peer-Reviewed Publication	1
13220	Randomized Pilot Study of Nimesulide Treatment in Alzheimer's Disease[24]	III	United States	Peer-Reviewed Publication	1
13222	Idebenone Treatment Fails to Slow Cognitive Decline in Alzheimer's Disease: A Multicentre, Randomised, Double-blind, Parallel, Placebo Comparison Study[25]	III	United States	Peer-Reviewed Publication	1
13229	Rofecoxib: No Effect on Alzheimer's Disease in a 1-year, Randomized, Blinded, Controlled Study[26]	III	United States	Peer-Reviewed Publication	1
13295	H2 Histamine Receptor Blockade in the Treatment of Alzheimer Disease: A Randomized, Double-Blind, Placebo-Controlled Trial of Nizatidine[27]	III	United States	Peer-Reviewed Publication	1
13308	A Randomized, Double-Blind, Placebo-Controlled Study of the Efficacy and Safety of Donepezil in Patients with Alzheimer's Disease in the Nursing Home Setting[28]	III	United States	Peer-Reviewed Publication	1
13440	A Multicenter, Randomized, Double-Blind, Placebo-Controlled, Flexible-Dose Study of Aripiprazole in the Treatment of Institutionalized Patients with Psychosis Associated with Dementia of the Alzheimer's Type[29]	III	United States	Peer-Reviewed Publication	1
13552	An Evaluation of the Long-Term Safety and Efficacy of Neramexane in Patients with Moderate to Severe Dementia of the Alzheimer's Type[30]	III	United States	No Publication	1
13558	A Randomized, Parallel-Group Pilot Study to Evaluate the Brain Effects and Efficacy of Exelon Compared to Aricept in Patients with Alzheimer's Disease with an Optional 12 Month Adjunctive Namenda Extension[31]	III	United States	No Publication	1

© 2026 Lin Z et al. *JAMA Network Open*.

13862	A Randomized, Double-Blind,Placebo-Controlled Evaluation of the Safety and Efficacy of Neramexane Monotherapy in Patients with Moderate to Severe Dementia of the Alzheimer's Type[32]	III	United States	Trial Report from Clinicaltrials.gov	1
13870	A Randomized, Parallel-Group Study to Evaluate the Efficacy of Trileptal Compared to Risperdal in Patients with Alzheimer's Dementia[33]	III	United States	No Publication	1
14182	A Large, Community-Based, Open-Label Trial of Donepezil in the Treatment of Alzheimer's Disease[34]	III	United States	Peer-Reviewed Publication	1
14877	Phase III Study of Citicoline for Alzheimer's Disease[35]	III	United States	No Publication	1
14978	A Head to Head Study of Donepezil, Rivastigmine, and Galantamine for the Treatment of Alzheimer's Disease[36]	III	United States	Conference Abstract	1
14981	Safety, Tolerability, and Caregiver's Impressions of Combination Therapy with Galantamine and Memantine for the Treatment of Alzheimer's Disease[37]	III	United States	Conference Abstract	1
14997	Donepezil Treatment Improves Cognitive Performance in Patients with Very Mild Alzheimer's Disease[38]	III	United States	Peer-Reviewed Publication	1
15031	An Open Label Study of Escitalopram (Lexapro) for the Treatment of Depression in Alzheimer's Disease (dAD)[39]	III	United States	Peer-Reviewed Publication	1
16530	A 12-Week, Open Label Trial of an Investigational Medication in the Treatment of Alzheimer's Disease[40]	III	United States	No Publication	1
19528	A Safety & Tolerability Study of Intramuscular Aripiprazole in Acutely Agitated Patients Diagnosed with Dementia[41]	III	United States	Peer-Reviewed Publication	1
20607	A Randomized, Double-Blind, Placebo-Controlled Evaluation of the Safety and Efficacy of Memantine in Non-Institutionalized Agitated Patients with Moderate to Severe Alzheimer's Disease[42]	III	United States	Trial Report from Clinicaltrials.gov	1

© 2026 Lin Z et al. *JAMA Network Open*.

21026	Correlates of Anticholinergic Activity in Patients with Dementia and Psychosis Treated with Risperidone or Olanzapine[43]	III	United States	Peer-Reviewed Publication	1
22468	Phase III, Multicenter, Randomized, Double Blind, Placebo Controlled Study of the Effect of Daily Treatment with MPC-7869 on Measures of Cognition, Activities of Daily Living and Global Function in Subjects with Mild Dementia of the Alzheimer's Type[44]	III	United States	Peer-Reviewed Publication	1
23825	A Long-Term Extension Study Evaluating the Safety and Tolerability of BID and QD Administration of Memantine in Patients with Mild to Moderate Dementia of the Alzheimer's Type-Phase A and B[45]	III	United States	Peer-Reviewed Publication	1
30214	A Randomized, Double-Blind, Placebo-Controlled Evaluation of the Safety and Efficacy of Memantine in Patients with Moderate to Severe Dementia of the Alzheimer's Type (A 24-week randomized, controlled trial of memantine in patients with moderate-to-severe Alzheimer disease) [46]	III	United States	Peer-Reviewed Publication	1
30217	A Randomized Double-Blind, Placebo-Controlled Evaluation of the Safety and Efficacy of Memantine in Patients with Moderate to Severe Dementia of Alzheimer's Type (Memantine Treatment in Patients With Moderate to Severe Alzheimer Disease Already Receiving Donepezil A Randomized Controlled Trial) [47]	III	United States	Peer-Reviewed Publication	1
30231	Double-Blind, Placebo-Controlled Evaluation of the Safety and Efficacy of Neramexane in Patients with Moderate to Severe Dementia of the Alzheimer's Disease[48]	III	United States	No Publication	1
30242	A Randomized, Double-Blind, Placebo-Controlled Evaluation of the Safety and Efficacy of Memantine in Patients with Mild to Moderate Dementia of the Alzheimer's Type (Memantine Treatment in Patients	III	United States	Peer-Reviewed Publication	1

© 2026 Lin Z et al. *JAMA Network Open*.

	with Mild to Moderate Alzheimers Disease Already Receiving a Cholinesterase Inhibitor: A Randomized, Double-Blind, Placebo-Controlled Trial)[49]				
30321	Randomized, Double-Blind, Placebo-Controlled Trial to Evaluate the Safety and Efficacy of Divalproex Sodium Therapy for Agitation in Nursing Home Residents with Probable or Possible Alzheimer's Disease[50]	III	United States	Peer-Reviewed Publication	1
32148	A Multicenter, Double Blind, Randomized Comparison of the Efficacy and Safety of Quetiapine Fumarate (Seroquel) and Placebo in the Treatment of Agitation Associated with Dementia[51]	III	United States	Peer-Reviewed Publication	1
32457	Long-Term Effects of Rivastigmine Treatment on the Need for Psychotropic Medications in Nursing Home Patients with Alzheimer's Disease[52]	III	United States	Peer-Reviewed Publication	1
34964	An Open-Label Evaluation of the Safety of Memantine in Patients with Moderate-to-Severe Dementia of the Alzheimer's Type[53]	III	United States	Conference Abstract	1
39718	The Effect of Memantine on Brain Structure and Chemistry in Alzheimer's Disease Patients: A Randomized, Placebo-Controlled, 52-Week Clinical Trial[54]	III	United States	Trial Report from Clinicaltrials.gov	1
41271	Preventing Cognitive Decline with Alternative Therapies[55]	III	United States	Peer-Reviewed Publication	1
41604	Efficacy of Donepezil in Mild Cognitive Impairment: A Randomized Placebo-Controlled Trial[56]	III	United States	Peer-Reviewed Publication	1
41817	A Long-Term Extension Study Evaluating the Safety and Tolerability of Four Memantine Dosing Regimens in Patients with Moderate to Severe Dementia of the Alzheimer's Type - Phases A and B[57]	III	United States	No Publication	1
41862	A Long-Term Extension Study Evaluating the Safety and Tolerability of Four Memantine Dosing Regimens	III	United States	No Publication	1

© 2026 Lin Z et al. *JAMA Network Open*.

	in Patients with Moderate to Severe Dementia of the Alzheimer's Type- Phase C[58]				
42139	A Long-Term Extension Study Evaluating the Safety and Tolerability of BID and QD Administration of Memantine in Patients with Mild to Moderate Dementia of the Alzheimer's Type- Phase C[59]	III	United States	No Publication	1
42145	Open Label Study Assessing the Safety and Efficacy of Combined Donepezil Plus Quetiapine in the Treatment of Agitated Demented Nursing Home Patients[60]	III	United States	No Publication	1
42540	Extension of a 28-Week, Randomized, Double-Blind, Placebo-Controlled Study of Memantine in Patients with Moderate to Severe Alzheimer Disease[61]	III	United States	Peer-Reviewed Publication	1
42700	Open-Label, Multicenter, Phase 3 Extension Study of the Safety and Efficacy of Donepezil in Patients with Alzheimer Disease[62]	III	United States	Peer-Reviewed Publication	1
47152	Placebo-Controlled Evaluation Of Galantamine In The Treatment Of Alzheimer's Disease: A Cardiac Safety Study.[63]	III	United States	Trial Report from Clinicaltrials.gov	1
52771	A Randomized, Double-Blind, Placebo-Controlled Evaluation of the Safety and Efficacy of Memantine in Patients with Mild to Moderate Dementia of the Alzheimer's Type, with A Long-Term, Open-Label Extension Study - Phase B[64]	III	United States	No Publication	1
52774	A Randomized, Double-Blind, Placebo-Controlled Evaluation of the Safety and Efficacy of Memantine In Patients with Mild to Moderate Dementia of the Alzheimer's Type, with A Long-Term, Open-Label Extension Study - Phase A[65]	III	United States	No Publication	1
58270	A Randomized Double-Blind Placebo-Controlled Trial of the Effects of Docosahexaenoic Acid (DHA) in Slowing the Progression of Alzheimer's Disease[66]	III	United States	Peer-Reviewed Publication	1

© 2026 Lin Z et al. *JAMA Network Open*.

60393	A Long-term Extension Study Evaluating the Safety and Tolerability of Four Memantine Dosing Regimens in Patients With Moderate to Severe Dementia of the Alzheimer's Type - Phase D[67]	III	United States	Trial Report from Pharmaceuticals	1
60890	A Long-Term Extension Study Evaluating the Safety and Tolerability of BID and QD Administration of Memantine in Patients with Mild to Moderate Dementia of the Alzheimer's Type-Phase D[68]	III	United States	No Publication	1
62512	A Single Center, Multi-Site, Randomized, Double-Blind, Placebo-Controlled Trial of Resveratrol with Glucose and Malate (RGM) to Slow the Progression of Alzheimer's Disease[69]	III	United States	Peer-Reviewed Publication	1
65044	A Multicenter, Randomized, Double-Blind Placebo-Controlled, Flexible-Dose Study of Aripiprazole in the Treatment of Institutionalized Patients with Psychosis Associated with Dementia of the Alzheimer's Type (Extension Phase of Protocol CN138005)[70]	III	United States	No Publication	1
65257	Rofecoxib for the Treatment of Mild Cognitive Impairment (MCI) and Prevention of Conversion to Alzheimer's Disease[71]	III	United States	Peer-Reviewed Publication	1
74871	Long-term Safety and Efficacy of Memantine Treatment in Moderate to Severe Alzheimer's Disease[72]	III	United States	Conference Abstract	1
77165	Testosterone Supplementation in Men with MCI[73]	III	United States	Peer-Reviewed Publication	1
77250	An Open-Label Extension Study Evaluating the Safety of Memantine in Patients with Moderate to Severe Dementia of the Alzheimer's type[74]	III	United States	Conference Abstract	1
93637	Treatment of Apathy in Alzheimer's disease (AD) with Modafinil[75]	III	United States	Peer-Reviewed Publication	1

© 2026 Lin Z et al. *JAMA Network Open*.

94359	A Randomized, Double-Blind, Placebo-Controlled Trial of Two Doses of Ginkgo Biloba Extract in Dementia of the Alzheimer's Type[76]	III	United States	Peer-Reviewed Publication	1
95528	Results of Next Day Crossover Study of Galantamine ER (Razadyne Er) to Rivastigmine Patch (Exelon Patch) in Alzheimer'sDisease Patients: A Two-Month Clinical Experience[77]	III	United States	Conference Abstract	1
95538	Results of Next Day Crossover Study of Donepezil (Aricept) to Rivastigmine Patch (Exelon Patch) in Alzheimer's Disease Patients: A Two-Month Clinical Experience[78]	III	United States	Conference Abstract	1
107748	Longitudinal Measure of IVIG Treatment Effect in Patients with Alzheimer's and Lewy Body Disease[79]	III	United States	Conference Abstract	1
112904	A 24 Week, Prospective, Randomized, Parallel-Group, Double-Blind, Multi-Center Study Comparing the Effects of Rivastigmine Patch 15 cm2 vs. Rivastigmine Patch 5 cm2 on Activities of Daily Living and CognitIon in Patients with Severe Dementia of the Alzheimer's Type (ACTION)[80]	III	United States	Peer-Reviewed Publication	1
129451	Mechanisms of Insulin Facilitation of Memory[81]	III	United States	Trial Report from Clinicaltrials.gov	1
140656	A Phase III / Pivotal Study of Rivastigmine, TDS for Alzheimer[82]	III	United States	No Publication	1
149121	A Randomized, Double-Blind, Placebo-Controlled Study to Evaluate the Effects of DHA on Cognitive Functions in the Elderly[83]	III	United States	Peer-Reviewed Publication	1
151549	Alzheimer's Disease Anti-Inflammatory Prevention Trial Follow-Up Study[84]	III	United States	Peer-Reviewed Publication	1
186159	A 20-month, Placebo-Controlled Research Study Evaluating an Investigational Drug for Alzheimer's Disease[85]	III	United States	No Publication	1
391940	A Phase III, Double Blind, Randomized, Placebo Controlled, Parallel Group, Multicenter Study of	III	United States	Trial Report from Pharmaceuticals	1

© 2026 Lin Z et al. *JAMA Network Open*.

	NE3107 in Subjects Who Have Mild to Moderate Alzheimer's Disease[86]				
399209	A Multicenter, Randomized, Double-Blind, Placebo-Controlled, Efficacy And Safety Study of PRN Dosing of BXCL501 Over A 12 Week Period In Subjects With Agitation Associated With Dementia[87]	III	United States	Trial Report from Clinicaltrials.gov	1
417081	A Phase 3, Open-Label, Parallel-Group, 2-Arm Study to Investigate Amyloid Plaque Clearance With Donanemab Compared With Aducanumab-avwa in Participants With Early Symptomatic Alzheimer's Disease[88]	III	United States	Trial Report from Clinicaltrials.gov	1

[a] The quality of evidence was assessed using the Quality Rating Scheme for Studies and Other Evidence (ratings of 1-5). Because all trials included in this review were classified as Phase 3 clinical trials in Trialtrove, each study was assigned a rating of 1.

© 2026 Lin Z et al. *JAMA Network Open*.

eReferences.

1. Singer C, Tractenberg RE, Kaye J, et al. A Multicenter, Placebo-controlled Trial of Melatonin for Sleep Disturbance in Alzheimer's Disease. *Sleep*. 2003;26(7):893-901. doi:10.1093/sleep/26.7.893

2. Snitz BE, O'Meara ES, Carlson MC, et al. Ginkgo biloba for Preventing Cognitive Decline in Older Adults: A Randomized Trial. *JAMA*. 2009;302(24):2663-2670. doi:10.1001/jama.2009.1913

3. Aisen PS, Schneider LS, Sano M, et al. High-Dose B Vitamin Supplementation and Cognitive Decline in Alzheimer Disease: A Randomized Controlled Trial. *JAMA*. 2008;300(15):1774-1783. doi:10.1001/jama.300.15.1774

4. Pollock Bruce G., Mulsant Benoit H., Rosen Jules, et al. Comparison of Citalopram, Perphenazine, and Placebo for the Acute Treatment of Psychosis and Behavioral Disturbances in Hospitalized, Demented Patients. *American Journal of Psychiatry*. 2002;159:460-465. doi:10.1176/appi.ajp.159.3.460

5. Sano M, Jacobs D, Andrews H, et al. A multi-center, randomized, double blind placebo-controlled trial of estrogens to prevent Alzheimer's disease and loss of memory in women: design and baseline characteristics. *Clinical Trials*. 2008;5(5):523-533. doi:10.1177/1740774508096313

6. Tariot PN, Solomon PR, Morris JC, et al. A 5-month, randomized, placebo-controlled trial of galantamine in AD. *Neurology*. 2000;54(12):2269-2276. doi:10.1212/WNL.54.12.2269

7. ADAPT Research Group. Alzheimer's Disease Anti-Inflammatory Prevention Trial: Design, methods, and baseline results. *Alzheimer's & Dementia*. 2009;5(2):93-104. doi:10.1016/j.jalz.2008.09.004

8. Aisen PS, Davis KL, Berg JD, et al. A randomized controlled trial of prednisone in Alzheimer's disease. *Neurology*. 2000;54(3):588-588. doi:10.1212/WNL.54.3.588

9. Teri L, Logsdon RG, Peskind E, et al. Treatment of agitation in AD. *Neurology*. 2000;55(9):1271-1278. doi:10.1212/WNL.55.9.1271

10. Mulnard RA, Cotman CW, Kawas C, et al. Estrogen Replacement Therapy for Treatment of Mild to Moderate Alzheimer DiseaseA Randomized Controlled Trial. *JAMA*. 2000;283(8):1007-1015. doi:10.1001/jama.283.8.1007

11. Sano M, Bell KL, Galasko D, et al. A randomized, double-blind, placebo-controlled trial of simvastatin to treat Alzheimer disease. *Neurology*. 2011;77(6):556-563. doi:10.1212/WNL.0b013e318228bf11

12. Mintzer J, Greenspan A, Caers I, et al. Risperidone in the Treatment of Psychosis of Alzheimer Disease: Results From a Prospective Clinical Trial. *The American Journal of Geriatric Psychiatry*. 2006;14(3):280-291. doi:10.1097/01.JGP.0000194643.63245.8c

© 2026 Lin Z et al. *JAMA Network Open.*

13. Small GW, Siddarth P, Silverman DHS, et al. Cognitive and Cerebral Metabolic Effects of Celecoxib Versus Placebo in People With Age-Related Memory Loss: Randomized Controlled Study. *The American Journal of Geriatric Psychiatry*. 2008;16(12):999-1009. doi:10.1097/JGP.0b013e31818cd3a4

14. Tariot PN, Schneider LS, Cummings J, et al. Chronic Divalproex Sodium to Attenuate Agitation and Clinical Progression of Alzheimer Disease. *Archives of General Psychiatry*. 2011;68(8):853-861. doi:10.1001/archgenpsychiatry.2011.72

15. Reisberg Barry, Doody Rachelle, Stöffler Albrecht, Schmitt Frederick, Ferris Steven, Möbius Hans Jörg. Memantine in Moderate-to-Severe Alzheimer's Disease. *New England Journal of Medicine*. 2003;348(14):1333-1341. doi:10.1056/NEJMoa013128

16. Sano Mary, Ernesto Christopher, Thomas Ronald G., et al. A Controlled Trial of Selegiline, Alpha-Tocopherol, or Both as Treatment for Alzheimer's Disease. *New England Journal of Medicine*. 1997;336(17):1216-1222. doi:10.1056/NEJM199704243361704

17. *A Phase III Study of ABP-124 for Alzheimer's Disease*. TrialTrove (Trial Record with No Publication).

18. Small G. *Anti-Inflammation in AD: PET Imaging Supplement*. clinicaltrials.gov; 2005. Accessed December 29, 2025. https://clinicaltrials.gov/study/NCT00065169

19. Cummings Jeffrey L., Schneider Lon, Tariot Pierre N., Kershaw Paul R., Yuan Weiying. Reduction of Behavioral Disturbances and Caregiver Distress by Galantamine in Patients With Alzheimer's Disease. *American Journal of Psychiatry*. 2004;161:532-538. doi:10.1176/appi.ajp.161.3.532

20. Scharre DW, Shiovitz T, Zhu Y, Amatniek J. One-week dose titration of extended release galantamine in patients with Alzheimer's disease. *Alzheimer's & Dementia*. 2007;4(1):30-37. doi:10.1016/j.jalz.2007.10.013

21. Peskind ER, Potkin SG, Pomara N, et al. Memantine Treatment in Mild to Moderate Alzheimer Disease: A 24-Week Randomized, Controlled Trial. *The American Journal of Geriatric Psychiatry*. 2006;14(8):704-715. doi:10.1097/01.JGP.0000224350.82719.83

22. Alzheimer's Association. *Galantamine in the Treatment of Moderate to Advanced Alzheimer's Disease: Clinical and Pharmacoeconomic Outcomes in the Assisted Living Environment*. Alzheimer's Association. Accessed December 29, 2025. http://www.abstractsonline.com/viewer/viewAbstract.asp?CKey={8A34B71C-6048-46DE-AA34-94962AC33502}&MKey={F38908E2-C78B-40E5-B4F4-9817BF853BD1}&AKey={50E1744A-0C52-45B2-BF85-2A798BF24E02}&SKey={BD4CCC85-1979-4B23-AE35-553338449AD1}

23. M. Gaudig, U. Richarz, J. Han, B. Van Baelen, B. Schauble. Effects of Galantamine in Alzheimers Disease: Double-blind Withdrawal Studies Evaluating Sustained Versus Interrupted Treatment. *Current Alzheimer Research*. 2011;8(7):771-780. doi:10.2174/156720511797633205

© 2026 Lin Z et al. *JAMA Network Open.*

24. Aisen PS, Schmeidler J, Pasinetti GM. Randomized pilot study of nimesulide treatment in Alzheimer's disease. *Neurology*. 2002;58(7):1050-1054. doi:10.1212/WNL.58.7.1050

25. Thal LJ, Grundman M, Berg J, et al. Idebenone treatment fails to slow cognitive decline in Alzheimer's disease. *Neurology*. 2003;61(11):1498-1502. doi:10.1212/01.WNL.0000096376.03678.C1

26. Reines SA, Block GA, Morris JC, et al. Rofecoxib: No effect on Alzheimer's disease in a 1-year, randomized, blinded, controlled study. *Neurology*. 2004;62(1):66-71. doi:10.1212/WNL.62.1.66

27. Carlson MC, Tschanz JT, Norton MC, Welsh-Bohmer K, Martin BK, Breitner JCS. H2 Histamine Receptor Blockade in the Treatment of Alzheimer Disease: A Randomized, Double-Blind, Placebo-Controlled Trial of Nizatidine. *Alzheimer Disease & Associated Disorders*. 2002;16(1). https://journals.lww.com/alzheimerjournal/fulltext/2002/01000/h2_histamine_receptor_blockade_in_the_treatment_of.4.aspx

28. Tariot PN, Cummings JL, Katz IR, et al. A Randomized, Double-Blind, Placebo-Controlled Study of the Efficacy and Safety of Donepezil in Patients with Alzheimer's Disease in the Nursing Home Setting. *Journal of the American Geriatrics Society*. 2005;49(12):1590-1599. doi:10.1111/j.1532-5415.2001.49266.x

29. Streim JE, Porsteinsson AP, Breder CD, et al. A Randomized, Double-Blind, Placebo-Controlled Study of Aripiprazole for the Treatment of Psychosis in Nursing Home Patients with Alzheimer Disease. *The American Journal of Geriatric Psychiatry*. 2008;16(7):537-550. doi:10.1097/JGP.0b013e318165db77

30. *An Evaluation of the Long-Term Safety and Efficacy of Neramexane in Patients with Moderate to Severe Dementia of the Alzheimer's Type*. TrialTrove (Trial Record with No Publication).

31. *A Randomized, Parallel-Group Pilot Study to Evaluate the Brain Effects and Efficacy of Exelon Compared to Aricept in Patients with Alzheimer's Disease with an Optional 12 Month Adjunctive Namenda Extension.* TrialTrove (Trial Record with No Publication).

32. Forest Laboratories. *A Randomized, Double-Blind,Placebo-Controlled Evaluation of the Safety and Efficacy of Neramexane Monotherapy in Patients With Moderate to Severe Dementia of the Alzheimer's Type*. clinicaltrials.gov; 2005. Accessed December 29, 2025. https://clinicaltrials.gov/study/NCT00090116

33. *A Randomized, Parallel-Group Study to Evaluate the Efficacy of Trileptal Compared to Risperdal in Patients with Alzheimer's Dementia*. TrialTrove (Trial Record with No Publication).

34. Relkin NR, Reichman WE, Orazem J, McRae T. A Large, Community-Based, Open-Label Trial of Donepezil in the Treatment of Alzheimer's Disease. *Dementia and Geriatric Cognitive Disorders*. 2003;16(1):15-24. doi:10.1159/000069988

© 2026 Lin Z et al. *JAMA Network Open.*

35. *Phase III Study of Citicoline for Alzheimer's Disease*. TrialTrove (Trial Record with No Publication).

36. Conference Abstract. *A Head to Head Study of Donepezil, Rivastigmine, and Galantamine for the Treatment of Alzheimer's Disease*. Alzheimer's Association; 2004. Accessed December 29, 2025. https://www.abstractsonline.com/viewer/viewAbstract.asp?CKey={3E7803E4-DCCE-4F90-B692-3551119FB711}&MKey={F38908E2-C78B-40E5-B4F4-9817BF853BD1}&AKey={50E1744A-0C52-45B2-BF85-2A798BF24E02}&SKey={BD4CCC85-1979-4B23-AE35-553338449AD1}

37. Conference Abstract. *Safety, Tolerability, and Caregiver's Impressions of Combination Therapy with Galantamine and Memantine for the Treatment of Alzheimer's Disease*. Alzheimer's Association; 2004. Accessed December 29, 2025. https://www.abstractsonline.com/viewer/viewAbstract.asp?CKey={B7A0303E-9EBF-40EE-AAD0-58297117B183}&MKey={F38908E2-C78B-40E5-B4F4-9817BF853BD1}&AKey={50E1744A-0C52-45B2-BF85-2A798BF24E02}&SKey={BD4CCC85-1979-4B23-AE35-553338449AD1}27

38. Seltzer B, Zolnouni P, Nunez M, et al. Efficacy of Donepezil in Early-Stage Alzheimer Disease: A Randomized Placebo-Controlled Trial. *Archives of Neurology*. 2004;61(12):1852-1856. doi:10.1001/archneur.61.12.1852

39. Rao V, Spiro JR, Rosenberg PB, Lee HB, Rosenblatt A, Lyketsos CG. An open-label study of escitalopram (Lexapro®) for the treatment of 'Depression of Alzheimer's disease' (dAD). *International Journal of Geriatric Psychiatry*. 2006;21(3):273-274. doi:10.1002/gps.1459

40. *A 12-Week, Open Label Trial of an Investigational Medication in the Treatment of Alzheimer's Disease*. TrialTrove (Trial Record with No Publication).

41. Rappaport SA, Marcus RN, Manos G, McQuade RD, Oren DA. A Randomized, Double-Blind, Placebo-Controlled Tolerability Study of Intramuscular Aripiprazole in Acutely Agitated Patients With Alzheimer's, Vascular, or Mixed Dementia. *Journal of the American Medical Directors Association*. 2009;10(1):21-27. doi:10.1016/j.jamda.2008.06.006

42. Forest Laboratories. *A Randomized, Double-Blind, Placebo-Controlled Evaluation of the Safety and Efficacy of Memantine in Non-Institutionalized Agitated Patients With Moderate to Severe Alzheimer's Disease*. clinicaltrials.gov; 2006. Accessed December 29, 2025. https://clinicaltrials.gov/study/NCT00097916

43. Mulsant BH, Gharabawi GM, Bossie CA, et al. Correlates of Anticholinergic Activity in Patients With Dementia and Psychosis Treated With Risperidone or Olanzapine. *J Clin Psychiatry*. 2004;65(12):1708-1714. doi:10.4088/JCP.v65n1217

44. Green RC, Schneider LS, Amato DA, et al. Effect of Tarenflurbil on Cognitive Decline and Activities of Daily Living in Patients With Mild Alzheimer Disease: A Randomized Controlled Trial. *JAMA*. 2009;302(23):2557-2564. doi:10.1001/jama.2009.1866

© 2026 Lin Z et al. *JAMA Network Open.*

45. Ott BR, Blake LM, Kagan E, Resnick M, for the Memantine MEM-MD-11AB Study Group. Open label, multicenter, 28-week extension study of the safety and tolerability of memantine in patients with mild to moderate Alzheimer's disease. *Journal of Neurology*. 2007;254(3):351-358. doi:10.1007/s00415-006-0374-x

46. van Dyck CH, Tariot PN, Meyers B, Malca Resnick E, for the Memantine MEM-MD-01 Study Group. A 24-week Randomized, Controlled Trial of Memantine in Patients With Moderate-to-severe Alzheimer Disease. *Alzheimer Disease & Associated Disorders*. 2007;21(2). https://journals.lww.com/alzheimerjournal/fulltext/2007/04000/a_24_week_randomized,_controlled_trial_of.12.aspx

47. Tariot PN, Farlow MR, Grossberg GT, et al. Memantine Treatment in Patients With Moderate to Severe Alzheimer Disease Already Receiving DonepezilA Randomized Controlled Trial. *JAMA*. 2004;291(3):317-324. doi:10.1001/jama.291.3.317

48. *Double-Blind, Placebo-Controlled Evaluation of the Safety and Efficacy of Neramexane in Patients with Moderate to Severe Dementia of the Alzheimer's Disease*. TrialTrove (Trial Record with No Publication).

49. Anton P. Porsteinsson, George T. Grossberg, Jacobo Mintzer, Jason T. Olin. Memantine Treatment in Patients with Mild to Moderate Alzheimers Disease Already Receiving a Cholinesterase Inhibitor: A Randomized, Double-Blind, Placebo-Controlled Trial. *Current Alzheimer Research*. 2008;5(1):83-89. doi:10.2174/156720508783884576

50. Tariot PN, Raman R, Jakimovich L, et al. Divalproex Sodium in Nursing Home Residents With Possible or Probable Alzheimer Disease Complicated by Agitation: A Randomized, Controlled Trial. *The American Journal of Geriatric Psychiatry*. 2005;13(11):942-949. doi:10.1097/00019442-200511000-00004

51. Zhong KX, Tariot PN, Mintzer J, Minkwitz MC, Devine NA. Quetiapine to Treat Agitation in Dementia: A Randomized, Double-Blind,Placebo-Controlled Study. *Current Alzheimer Research*. 2007;4(1):81-93. doi:10.2174/156720507779939805

52. Edwards K, Koumaras B, Chen M, Gunay I, Mirski D, Rivastigmine Nursing Home Study Team. Long-Term Effects of Rivastigmine Treatment on the Need for Psychotropic Medications in Nursing Home Patients with Alzheimer's Disease. *Clinical Drug Investigation*. 2005;25(8):507-515. doi:10.2165/00044011-200525080-00003

53. Conference Abstract. *An Open-Label Evaluation of the Safety of Memantine in Patients with Moderate-to-Severe Dementia of the Alzheimer's Type*. Annual American Academy of Neurology Meeting; 2010.

54. Yesavage JA. *The Effect of Memantine on Brain Structure and Chemistry in Alzheimer's Disease Patients: A Randomized, Placebo-Controlled, 52-Week Clinical Trial*. clinicaltrials.gov; 2010. Accessed December 29, 2025. https://clinicaltrials.gov/study/NCT00255086

55. Dodge HH, Zitzelberger T, Oken BS, Howieson D, Kaye J. A randomized placebo-controlled trial of Ginkgo biloba for the prevention of cognitive decline. *Neurology*. 2008;70(19_part_2):1809-1817. doi:10.1212/01.wnl.0000303814.13509.db

56. Salloway S, Ferris S, Kluger A, et al. Efficacy of donepezil in mild cognitive impairment. *Neurology*. 2004;63(4):651-657. doi:10.1212/01.WNL.0000134664.80320.92

57. *A Long-Term Extension Study Evaluating the Safety and Tolerability of Four Memantine Dosing Regimens in Patients with Moderate to Severe Dementia of the Alzheimer's Type - Phases A and B*. TrialTrove (Trial Record with No Publication).

58. *A Long-Term Extension Study Evaluating the Safety and Tolerability of Four Memantine Dosing Regimens in Patients with Moderate to Severe Dementia of the Alzheimer's Type-Phase C*. TrialTrove (Trial Record with No Publication).

59. *A Long-Term Extension Study Evaluating the Safety and Tolerability of BID and QD Administration of Memantine in Patients with Mild to Moderate Dementia of the Alzheimer's Type- Phase C*. TrialTrove (Trial Record with No Publication).

60. *Open Label Study Assessing the Safety and Efficacy of Combined Donepezil Plus Quetiapine in the Treatment of Agitated Demented Nursing Home Patients*. TrialTrove (Trial Record with No Publication).

61. Reisberg B, Doody R, Stöffler A, Schmitt F, Ferris S, Möbius HJ. A 24-Week Open-Label Extension Study of Memantine in Moderate to Severe Alzheimer Disease. *Archives of Neurology*. 2006;63(1):49-54. doi:10.1001/archneur.63.1.49

62. Doody RS, Geldmacher DS, Gordon B, Perdomo CA, Pratt RD, for the Donepezil Study Group. Open-Label, Multicenter, Phase 3 Extension Study of the Safety and Efficacy of Donepezil in Patients With Alzheimer Disease. *Archives of Neurology*. 2001;58(3):427-433. doi:10.1001/archneur.58.3.427

63. Johnson & Johnson Pharmaceutical Research & Development, L.L.C. *Placebo-Controlled Evaluation of Galantamine in the Treatment of Alzheimer's Disease: A Cardiac Safety Study.* clinicaltrials.gov. Accessed December 29, 2025. https://clinicaltrials.gov/study/NCT00309725

64. *A Randomized, Double-Blind, Placebo-Controlled Evaluation of the Safety and Efficacy of Memantine in Patients with Mild to Moderate Dementia of the Alzheimer's Type, with A Long-Term, Open-Label Extension Study - Phase B*. TrialTrove (Trial Record with No Publication).

65. *A Randomized, Double-Blind, Placebo-Controlled Evaluation of the Safety and Efficacy of Memantine In Patients with Mild to Moderate Dementia of the Alzheimer's Type, with A Long-Term, Open-Label Extension Study - Phase A*. TrialTrove (Trial Record with No Publication).

© 2026 Lin Z et al. *JAMA Network Open.*

66. Quinn JF, Raman R, Thomas RG, et al. Docosahexaenoic Acid Supplementation and Cognitive Decline in Alzheimer Disease: A Randomized Trial. *JAMA*. 2010;304(17):1903-1911. doi:10.1001/jama.2010.1510

67. Forest Laboratories, Inc. *A Long-Term Extension Study Evaluating the Safety and Tolerability of Four Memantine Dosing Regimens in Patients With Moderate to Severe Dementia of the Alzheimer's Type - Phase D*. Forest Laboratories, Inc.; 2006.

68. *A Long-Term Extension Study Evaluating the Safety and Tolerability of BID and QD Administration of Memantine in Patients with Mild to Moderate Dementia of the Alzheimer's Type-Phase D*. TrialTrove (Trial Record with No Publication).

69. Zhu CW, Grossman H, Neugroschl J, et al. A randomized, double-blind, placebo-controlled trial of resveratrol with glucose and malate (RGM) to slow the progression of Alzheimer's disease: A pilot study. *Alzheimer's & Dementia: Translational Research & Clinical Interventions*. 2018;4(1):609-616. doi:10.1016/j.trci.2018.09.009

70. *A Multicenter, Randomized, Double-Blind Placebo-Controlled, Flexible-Dose Study of Aripiprazole in the Treatment of Institutionalized Patients with Psychosis Associated with Dementia of the Alzheimer's Type (Extension Phase of Protocol CN138005)*. TrialTrove (Trial Record with No Publication).

71. Thal LJ, Ferris SH, Kirby L, et al. A Randomized, Double-Blind, Study of Rofecoxib in Patients with Mild Cognitive Impairment. *Neuropsychopharmacology*. 2005;30(6):1204-1215. doi:10.1038/sj.npp.1300690

72. Aupperle P, Tariot P, Safirstein B, Graham SM, Lee G, Tocco M. Long-term safety and efficacy of memantine treatment in moderate to severe Alzheimer's disease: Results from a three-year trial. *Journal of the American Medical Directors Association*. 2007;8(3):B9. doi:10.1016/j.jamda.2007.01.050

73. Cherrier MM, Anderson K, Shofer J, Millard S, Matsumoto AM. Testosterone Treatment of Men With Mild Cognitive Impairment and Low Testosterone Levels. *Am J Alzheimers Dis Other Demen*. 2014;30(4):421-430. doi:10.1177/1533317514556874

74. Perhach J, Graham S. P4-454: A long-term, open-label extension study evaluating the safety of extended-release memantine (28 mg) in patients with moderate to severe Alzheimer's disease. *Alzheimer's & Dementia*. 2011;7(4S_Part_25):e70-e70. doi:10.1016/j.jalz.2011.09.149

75. Frakey LL, Salloway S, Buelow M, Malloy P. A Randomized, Double-Blind, Placebo-Controlled Trial of Modafinil for the Treatment of Apathy in Individuals With Mild-to-Moderate Alzheimer's Disease. *J Clin Psychiatry*. 2012;73(06):796-801. doi:10.4088/JCP.10m06708

76. Lon S. Schneider, Steven T. DeKosky, Martin R. Farlow, Pierre N. Tariot, Robert Hoerr, Meinhard Kieser. A Randomized, Double-Blind, Placebo-Controlled Trial of Two Doses of

© 2026 Lin Z et al. *JAMA Network Open.*

Ginkgo Biloba Extract in Dementia of the Alzheimers Type. *Current Alzheimer Research*. 2005;2(5):541-551. doi:10.2174/156720505774932287

77. Shua-Haim JR, Yap C, Kretov A, Lee P, Patel S. P2-410: Results of next day crossover study of galantamine ER (Razadyne ER) to rivastigmine patch (Exelon Patch) in Alzheimer's disease patients: A two-month clinical experience. *Alzheimer's & Dementia*. 2008;4(4S_Part_15):T493-T494. doi:10.1016/j.jalz.2008.05.1489

78. Shua-Haim JR, Yap C, Kretov A, Lee P. P2-408: Results of next day crossover study of donepezil (Aricept) to rivastigmine patch (Exelon Patch) in Alzheimer's disease patients: A two-month clinical experience. *Alzheimer's & Dementia*. 2008;4(4S_Part_15):T493-T493. doi:10.1016/j.jalz.2008.05.1487

79. Shankle WR, Hara J. P3-297: Longitudinal measure of IVIG treatment effect in patients with Alzheimer's and Lewy Body disease. *Alzheimer's & Dementia*. 2009;5(4S_Part_14):P430-P430. doi:10.1016/j.jalz.2009.04.968

80. Farlow MR, Grossberg GT, Sadowsky CH, Meng X, Somogyi M. aA 24-Week, Randomized, Controlled Trial of Rivastigmine Patch 13.3 mg/24 h Versus 4.6 mg/24 h in Severe Alzheimer's Dementia. *CNS Neurosci Ther*. 2013;19(10):745-752. doi:10.1111/cns.12158

81. Haley AP. *Mechanisms of Insulin Facilitation of Memory*. clinicaltrials.gov; 2014. Accessed December 29, 2025. https://clinicaltrials.gov/study/NCT01145482

82. *A Phase III / Pivotal Study of Rivastigmine, TDS for Alzheimer*. TrialTrove (Trial Record with No Publication).

83. Yurko-Mauro K, McCarthy D, Rom D, et al. Beneficial effects of docosahexaenoic acid on cognition in age-related cognitive decline. *Alzheimer's & Dementia*. 2010;6(6):456-464. doi:10.1016/j.jalz.2010.01.013

84. The Alzheimer's Disease Anti-inflammatory Prevention Trial Research Group. Results of a follow-up study to the randomized Alzheimer's Disease Anti-inflammatory Prevention Trial (ADAPT). *Alzheimer's & Dementia*. 2013;9(6):714-723. doi:10.1016/j.jalz.2012.11.012

85. *A 20-Month, Placebo-Controlled Research Study Evaluating an Investigational Drug for Alzheimer's Disease*. TrialTrove (Trial Record with No Publication).

86. BioVie. BioVie Announces Completion of Last Patient Treatment Visit in Phase 3 Trial of NE3107 in Mild to Moderate Alzheimer's Disease - BioSpace. Accessed December 29, 2025. https://www.biospace.com/biovie-announces-completion-of-last-patient-treatment-visit-in-phase-3-trial-of-ne3107-in-mild-to-moderate-alzheimer-s-disease

87. BioXcel Therapeutics Inc. *A Multicenter, Randomized, Double-Blind, Placebo-Controlled, Efficacy And Safety Study of PRN Dosing of BXCL501 Over A 12 Week Period In Subjects With Agitation Associated With Dementia*. clinicaltrials.gov; 2023. Accessed December 29, 2025. https://clinicaltrials.gov/study/NCT05271552

© 2026 Lin Z et al. *JAMA Network Open.*

88. Eli Lilly and Company. *A Phase 3, Open-Label, Parallel-Group, 2-Arm Study to Investigate Amyloid Plaque Clearance With Donanemab Compared With Aducanumab-Avwa in Participants With Early Symptomatic Alzheimer's Disease*. clinicaltrials.gov; 2024. Accessed December 29, 2025. https://clinicaltrials.gov/study/NCT05108922

© 2026 Lin Z et al. *JAMA Network Open.*
