# Supplementary material for: Racial and Ethnic Reporting and Representation in US Alzheimer Clinical Trials: A Systematic Review
Source: JAMA Netw Open. 2026 Mar 27;9(3):e262427. doi: 10.1001/jamanetworkopen.2026.2427 (PMC13032159; doi:10.1001/jamanetworkopen.2026.2427)
Supplement: Supplement 2. — Data Sharing Statement [file jamanetwopen-e262427-s002.pdf]

Data Sharing Statement

Lin. Racial and Ethnic Reporting and Representation in US Alzheimer Clinical Trials. *JAMA Netw Open*. Published March 27, 2026. doi:10.1001/jamanetworkopen.2026.2427

Data
Data available: Yes
Data types: Data (not involving human participants)
How to access data: Data supporting the findings of the study are available from the corresponding author upon reasonable request.
When available: With publication

Supporting Documents
Document types: None

Additional Information
Who can access the data: Data supporting the findings of the study are available from the corresponding author upon reasonable request.
Types of analyses: for a specified purpose
Mechanisms of data availability: with investigator support
